# Untangling the role of social relationships in the association between caregiver burden and caregiver health: an observational study exploring three coping models of the stress process paradigm

**DOI:** 10.1186/s12889-022-14127-3

**Published:** 2022-09-13

**Authors:** Hannah Tough, Martin W. G. Brinkhof, Christine Fekete

**Affiliations:** 1 Swiss Paraplegic Research, Guido A. Zäch Strasse 4, 6207 Nottwil, Switzerland; 2 Department of Health Sciences and Medicine, University of Lucerne, Lucerne, Switzerland

**Keywords:** Caregivers, Social support, Social environment, Spinal cord injury, Informal care, Caregiver burden

## Abstract

**Background:**

Caregivers health is often at risk due to the detrimental effects of caregiver burden. It is therefore vital to identify strategies and resources, which ensure the safeguarding of caregivers' health, whilst also enabling caregivers to continue providing high quality long-term care to care-receivers. The objective of this study is therefore to examine the moderating and mediating role of different social relationship constructs (social networks, social support, relationship quality, and loneliness) in the relationship between subjective caregiver burden and health, by exploring different coping models of the stress process paradigm, namely the *stress buffering*, social *deterioration* and *counteractive* models.

**Methods:**

Longitudinal survey data from 133 couples of caregiving romantic partners and persons with spinal cord injury, living in Switzerland were used. We employed multivariable regression analysis with the inclusion of interaction terms to explore moderation effects of social relationships (i.e. stress buffering model), and path analysis to explore mediation effects (i.e. social deterioration vs. counteractive model) of social relationships on the association between subjective caregiver burden and health. Health was operationalised using the following outcomes: mental health, vitality, bodily pain and general health.

**Results:**

Social support and relationship quality were found to buffer the negative effects of subjective caregiver burden on mental health. Mediating effects of social relationships were observed for mental health (indirect effect -0.25, -0.42- -0.08) and vitality (indirect effect -0.20, -0.37- -0.03), providing support for the *deterioration model*. Loneliness was found to be a particularly important construct on the pathway from caregiver burden to health.

**Conclusion:**

Our study highlights the potential of social support and relationship quality to override the negative consequences of caregiver burden on mental health and vitality. Our evidence thus supports the advance of interventions that seek to improve qualitative aspects of social relationships, especially in caregivers experiencing a high subjective caregiver burden.

**Supplementary Information:**

The online version contains supplementary material available at 10.1186/s12889-022-14127-3.

## Background

Informal caregivers who appraise their situation as emotionally and psychologically stressful are often vulnerable to subjective caregiver burden, which may have detrimental effects on physical and mental health [1–3]. Informal care describes the non-professional and unpaid care provided to persons with long-term care needs by family members, friends, neighbours, or other persons [4], and often covers a large proportion of the caregiving needs of the care-receiver [5, 6]. Ensuring that the burden of caregiving is reduced in this population is of upmost importance; primarily for the negative impact which subjective burden has on caregivers' health, but also for the wide-ranging individual and societal benefits resulting from the long-term care provided by informal caregivers. The individual benefit refers to the continuous high-quality and personalised care provided by informal caregivers [7] and societal benefits mainly concern the contributions in terms of reduced costs for the health care system [8, 9]. It is therefore vital to identify strategies and resources, which protect caregivers' health.

Social relationships are potentially modifiable resources that can promote caregivers health [10, 11] and may protect informal caregivers from the harmful effects of caregiver burden on physical and mental health [12–15]. More specifically, a study in informal caregivers of persons with traumatic brain injury showed that social support moderated the harmful effect of psychological distress on family functioning [12]. Another study in the context of Alzheimer’s disease found that provisioning od social support to caregivers moderated the association between caregiver distress and resilience, indicating that the negative effect of caregiver distress on resilience was reduced in caregivers with high social support [14]. This conclusion was supported by research in informal caregivers of persons with early-stage dementia [15].

Broadly speaking, social relationships describe any interpersonal interaction within a social network. Social relationships include quantitative aspects, such as frequency of social contact or network size, and qualitative aspects, such as perceived social support or the quality and utility of social contacts [16]. In this study, quantitative aspects were captured with social network inclusion, i.e., marital status, participation in church or community organizations, and the number of close friends and relatives. Qualitative measures used in this study include availability and quality of emotional and tangible social support would be available in case needed, the partner relationship quality in terms of its supportiveness and depth, and the frequency of feelings of loneliness. Yet, putative and observed associations of social relationships with caregiver health typically involve complex chains of direct and indirect effects, which may obscure potential targets of intervention. Previous research has identified the link between social relationships and caregiver burden [17–19], and social relationships and health [10], but few have connected these pathways together. An empirical evaluation of these pathways is warranted as to provide further insight into the potential leverage and thereby optimal targeting of social support interventions. …

**Fig. 1:**
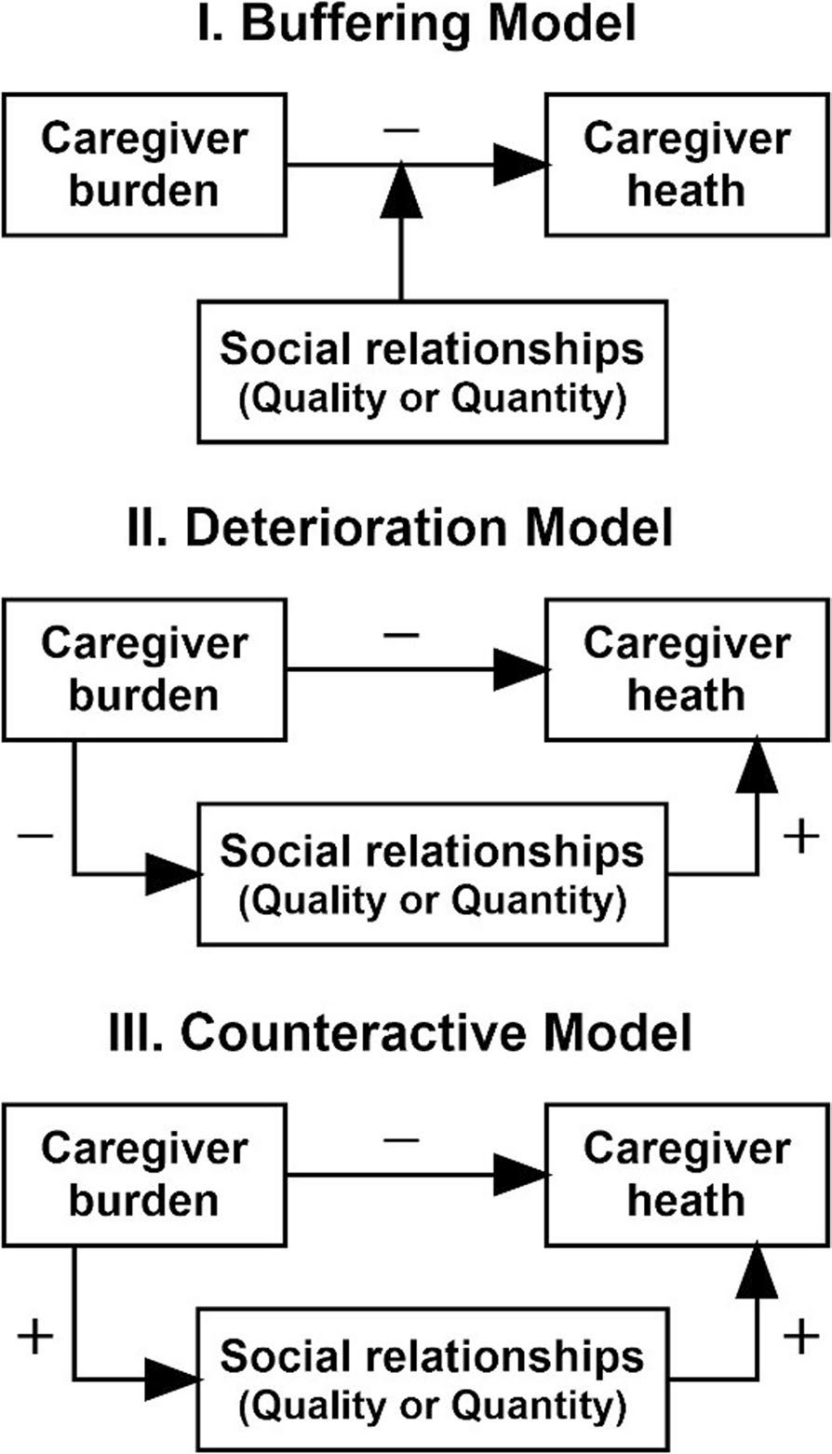
Coping models of the stress process paradigm

The present empirical study considers the three leading contemporary theoretical models for describing the role of social relationships in the association between caregiver burden and health (see Fig. 1). Firstly, and most prominently, the *buffering model* proposes the moderating effect of social relationships on the negative association between caregiver burden and health. There is as yet, no evidence supporting this model, however several studies have demonstrated how social support buffers the negative effects of objective caregiver burden (i.e. the time invested in caregiving) on subjective burden, and on caregiver distress and depression [12, 15, 20–23]. It is thought that social support and good quality relationships, signified as trusting, reciprocal and supportive, provide resources which aid caregivers to appraise their situation as less stressful, but also provide practical assistance to alleviate the caregiving burden and facilitate healthy behaviours [13, 24, 25]. Although there is evidence suggesting that social support protects caregivers from experiencing caregiver burden [22, 26], it remains unclear whether social support, or social relationships more broadly, can impede the negative effects of subjective caregiver burden on health. Secondly, the *deterioration model* proposes that the subjective caregiver burden has a damaging effect on relationships and therefore additionally negatively affects health. For example, evidence suggests that the additional role of caregiving in a partner relationship results in strain and tension leading to the degradation of the relationship quality [27, 28]. Thirdly, the *counteractive model* presents a contrasting hypothesis to the deterioration model and suggests that stressful situations cause individuals to utilise available resources from their existing social networks [29]. Applied to the case of caregiver burden, it supposes that the caregiving situation may motivate individuals to mobilise their existing resources, resulting in a higher level of perceived and received social support leading to beneficial effects on health. However, evidence for the counteractive model in the caregiving setting is unavailable yet.

In order to better understand the associations between stressors (i.e., subjective caregiver burden), resources (i.e., social relationships) and caregiver health, this study explores the presented models in the context of the caregiving romantic partners of persons with spinal cord injury (SCI). SCI offers an informative case in point, not only as it often leads to dependency on informal caregivers but also as it is a condition, which can occur at any point in life. Caregiving romantic partners of persons with SCI are often of employable age, which is in contrast to the vast majority of caregiving research mainly focusing on elderly populations [12, 14, 15]. In this study, we include couples who indicated being in a romantic partnership, irrespective of marital status or whether living together or not, and in which one partner takes over any informal caregiving activities. The objective of this study is therefore to examine the moderating or mediating role of different social relationship constructs (quantitative aspects: social networks; qualitative aspects: emotional and tangible social support, partner relationship quality, and feelings of loneliness) in the relationship between subjective caregiver burden and health in caregiving romantic partners of persons with SCI by evaluating and comparing the empirical support provided by our data for three coping models of the stress process paradigm (Fig. 1). The specific aims are 1) to test the *buffering model* by testing whether social relationships buffer or moderate the negative effects of subjective caregiver burden on caregiver health and 2) to test the two contrasting models of the *social deterioration* and the *counteractive model* by evaluating the mediating role of social relationships in the relationship between subjective caregiver burden and caregiver health.

## Methods

### Study design

Pro-WELL is a longitudinal community survey with three measurement waves (baseline; month 6; month 12) with the main objective to investigate the psychosocial determinants of wellbeing in persons with SCI and their caregiving romantic partners who are involved in caregiving duties. The survey has informed several studies on caregiver health, and on the social determinants of health and wellbeing in couples coping with disability and this paper is therefore one of a series. This analysis utilized longitudinal data from caregiving romantic partners of persons with SCI (*n* = 133). The baseline assessment was carried out between May 2015 and January 2016, and data were collected by means of standardized telephone interviews, paper–pencil or online questionnaires [30]. The study protocol and all measurements were approved by the Ethical Committee of Northwest and Central Switzerland (document EKNZ 2014–285). Regulations concerning informed consent and data protection were strictly observed and all participants signed an informed consent form. The study was conducted in accordance with the declaration of Helsinki.

### Sampling frame and participants

**Fig. 2:**
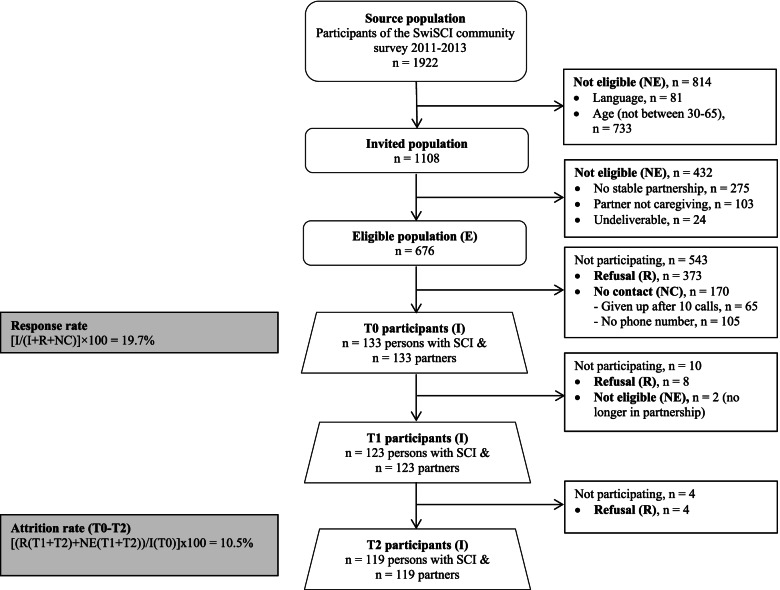
Source population and participation status of eligible persons

The pro-WELL study is a nested study that collected new data among participants of the first community survey of the Swiss Spinal Cord Injury Cohort Study (SwiSCI) [30]. This sampling frame included a representative population of 1922 persons aged over 16 years with traumatic or non-traumatic SCI living in Switzerland. Of the 1922 SwiSCI participants, 676 persons were eligible for the pro-WELL study. The pro-WELL study only included couples in a romantic partnership consisting of a person with SCI who indicated having a romantic partner which takes part in caregiving duties. The civil status in the partnership was not defined as inclusion or exclusion criterion and legally married as well as engaged or unmarried couples were included, and the romantic partner status was self-defined. A total number of 133 persons with SCI and their caregiving romantic partners participated in the baseline assessment, implying an overall response rate of 19.7%. Non-response bias, assessed by comparing the distribution of key sociodemographic and injury characteristic variables of the SCI participant in pro-WELL to the those of the SwiSCI source population, was shown to be negligible [30]. Finally, longitudinal study adherence of pro-WELL was respectable at 92% (123 couples) at 6 months, and 89% (119 couples) at 12 months (see Fig. 2). Further details on inclusion criteria, recruitment outcomes, participation rates, and non-response are reported in the pro-WELL cohort profile [30].

### Measures

#### Predictor: caregiver burden

*Subjective caregiver burden* was assessed using the 12-item Zarit Burden Interview (ZBI) short form, which captures personal feelings of strain resulting from the caregiving role [31, 32]. For example, participants were asked whether they experienced feelings of anger or strain, and whether the caregiving role had impinged on other areas of their lives. The five-point response scale includes the options never; rarely; sometimes; frequently; or nearly always. A sum score ranging from 0–48 was calculated.

#### Potential mediators/moderators: social relationships

The three coping models of the stress process paradigm (Fig. 1) imply a mediating or moderating role of social relationships. The Buffering Model (I) foresees a moderating role, that is, social relationships influence the relationship between caregiver burden and caregiver health, but are not as such on the causal pathway. In contrast, the Deterioration Model (II) and the Counteractive Model (III) postulate a mediating role, presuming that social relationships are on the causal pathway between caregiver burden and caregiver health. When considering the social relationships, mediation may emerge as full, if no remaining direct effect between caregiver burden and caregiver health is detectable, or rather partial, in case an direct as well as indirect effect materialize.

### Quantitative

*Social networks* were measured using five items from the Social Network Index (SNI) [33]. The SNI is a composite measure of four types of social connection: marital status (married = 1; not married = 0); church group membership (yes = 1; no = 0); membership in other community organisations (yes = 1; no = 0), and sociability (high = 1; low = 0). The latter concept included two items assessing the number of close friends and relatives. Persons indicating having at least three friends or relatives with which they were closely in touch were coded as 1, whereas persons indicating a lower number were coded as 0. In summary, each of the four types of social connection was scored with either 0 or 1 to signify whether an individual had access to this type of social connection or not and a sum score ranging from 0–4 was built.

### Qualitative

#### Social support

Emotional and tangible aspects of perceived social support were measured with items from the Swiss Health Survey 2012. Emotional support was assessed using the question “Among the people you are close to, do you have somebody who you can always talk to about personal problems?”, response options included: none, one person, or more persons [5]. Tangible support was assessed in the following areas: housework, health issues, financial issues, activities of daily living (in persons with SCI only), and caregiving (in caregiving romantic partners only). Response options for each area were: none, one person, or more persons. Scales for emotional and tangible support were added together for this study in order to give an overall sum score ranging from 0–10.

#### Relationship quality

Quality of partner relationship was assessed using items from the social support and depth subscales of the Quality of Relationship Inventory (QRI) which evaluated the meaningfulness and the positive role of the partnership, the extent to which one could turn to one’s partner for support, and the responsibility or need one felt for their partner.). The eight items were rated on a 4-point Likert scale, resulting in a sum score ranging from 0–24 [34].

#### Loneliness

Three items from the Revised UCLA loneliness scale [35] were used to capture the frequency of subjective feelings of loneliness (0 = almost never; 1 = sometimes; 2 = often), with a score ranging from 0–6. Due to the skewed distribution of responses, we dichotomised the score in order to discriminate persons who sometimes or often felt lonely from persons who never felt lonely (0 = never lonely; 1–6 = sometimes or often lonely).

### Outcome: caregiver health

In order explore different dimensions of health, five items of the SF-12 were utilised as indicators of caregiver health, namely items relating to mental health, vitality, bodily pain and general health. The SF-12 is the 12 item version of the previously developed 36-item version SF-36 and is widely used as a brief, time-saving version for health surveys [36]. The SF-36 was initially developed and validated as a generic instrument for measuring health status in the Medical Outcomes Study and consists of the eight domains including physical functioning; role restrictions due to physical health; bodily pain; general health; vitality; social functioning; role restrictions due to mental health; and mental health [37]. The SF-12 exploits substantially fewer items to effectively represent the same domains as evidenced by the very high correlation of its summary physical and mental health scores with those of the SF-36 [36, 38]. General health was assessed with one SF-12 item asking participants how they would rate their general health (0 = very poor, 4 = very good). The SF-12 measures mental health with two items on the frequency of mood states during the past 4 weeks, and vitality with one item on the frequency of feeling energetic during the past four weeks (response options for both, mental health and vitality: 0 = never, 5 = always). Pain was assessed with one SF-12 item on how much bodily pain was experienced during the past four weeks (0 = not at all, 5 = extreme). In line with SF-36 scoring recommendations, each subscale was transformed to a 0–100 scale, and then standardised to be comparable to a sample from the general population in order to complete the "norm-based scoring" [39].

### Confounders and colliders

The identification of potential confounders was informed by current evidence and by directed acyclic graphs (DAGs; www.dagitty.net) [3, 5, 12, 14, 15]. Utilising DAGs enables the identification of ‘true’ confounders which can subsequently be tested and validated in bivariable analysis [40]. Age, gender, financial hardship, employment status (having paid work vs. not having paid work), lesion severity of the care-receiver (para/tetraplegic, incomplete/complete lesion) and length of caregiving in years were identified as relevant confounders and were therefore included into multivariate models. Financial hardship was assessed with an item asking participants how they evaluate the availability of financial resources on a 5-point scale ranging from ‘very scarce’ to ‘lasts very well’.

We identified survey response mode (via telephone interview, paper–pencil or online) as a potential collider variable, as likely determined by both the predictor variable (subjective caregiver burden) and the outcome variable (caregiver health). To avoid biased inference regarding the association between caregiver burden and caregiver health, we thus refrained from controlling for questionnaire response mode in statistical analyses.

### Statistical analysis

Analyses were conducted using STATA version 16.1 for Windows (College Station, TX, USA). Distribution of predictor, mediator, moderator and outcome variables were described and dyadic concordance was assessed using multi-level models to compute within- and between-dyad variation. Intra-class correlations (ICCs) were evaluated to investigate how similar different variables were within dyads, with values closer to 1 indicating higher correlation within the dyad. Those descriptive analyses were performed with crude baseline data, excluding all cases with missing values. In order to explore moderation and mediation models, longitudinal data was utilised. As caregiver burden was only measured at baseline, we utilised data from specific time points. Measures of subjective caregiver burden and measures of confounders were taken at baseline (T0), social relationships at baseline (T0) for moderation analysis and at 6 months (T1) for mediation analysis and caregiver health at 12 months (T2).

#### Moderation analysis

To explore interaction effects of social relationship constructs and caregiver burden in predicting caregiver health, we computed a series of hierarchical regression models. The moderator and predictor variables were mean-centered prior to analysis as to minimize the risk of multicollinearity. In each regression model, one of the caregiver health indicators was utilised as the dependant variable and the mean-centered predictor and moderator variables were entered first alone and then with their corresponding interaction term. Each potential moderator variable (i.e. social relationship construct) was introduced independently of the other potential moderator variables. Therefore, Model 1 represents an unadjusted analysis of the main effects of both, predictors and potential moderators. In Model 2, we entered the interaction terms as a third independent variable and in Model 3 in order to address potential confounding, the confounders age, sex, language region, lesion severity of the care-receiver, caregiving duration in years, employment status and financial hardship were also entered. Interaction terms were evaluated by testing the coefficient of the product term itself, and interaction terms with *p* < 0.05 were further graphically examined to support the interpretation of results. To visualize interaction terms over the observed range of the interdependent variables, marginal predictions of the regression model were derived and plotted using a two-by-two contour plot. To account for potential non-response bias resulting from item non-response in predictor and control variables, multiply imputed data that were derived by multiple imputation using chained equations (MICE) were used for regression models, unimputed data was used for descriptive analysis (results in Table 1), giving a complete case analysis sample size of 94[41]. Item missingness was assumed to be missing completely at random and therefore the multiple imputation should effectively assist to reduce non-response bias. Selection bias due to unit non-response has been shown to be negligible and was therefore not accounted for in data analysis [30].

**Table 1:** Characteristics of the pro-WELL sample (*N* = 266)

		**Caregiving romantic partners (*N* = 133)**		**Care-receivers, persons with SCI (*N* = 133)**		**ICC (95% CI)**
	**Characteristic [n missing values caregiving romantic partners, care-receivers]**	**n (%)**	**Mean (SD); Median (IQR)**	**n (%)**	**Mean (SD); Median (IQR)**	**Within-dyad comparison**
**Baseline**	**Sociodemographic characteristics**					
	Age (in years) [0,0]		50.2 (10.1); 52.0 (16.0)		51.7 (9.4); 53.0 (16.0)	0.79 (0.72, 0.84)
	Female gender [0,0]	98 (73.7)		35 (26.3)		-
	Education (in years) [2, 7]		14.0 (3.1); 13.5 (4.0)		13.9 (3.2); 13.0 (4.0)	0.27 (0.14, 0.46)
	Financial hardship	39 (33.1)		38 (31.9)		0.55 (0.32, 0.76)
	Paid employment [0,0]	94 (70.7)		79 (59.4)		0.27 (0.09, 0.57)
	**Lesion characteristics**					
	Lesion severity [2]					
	Incomplete paraplegia	- -		45 (34.4)		- -
	Complete paraplegia	- -		49 (37.4)		- -
	Incomplete tetraplegia	- -		24 (18.3)		- -
	Complete tetraplegia	- -		13 (9.9)		- -
	Aetiology [3]					
	Traumatic			109 (83.8)		
	Non-traumatic			21 (16.2)		
	**Objective caregiver burden**					
	Duration of daily care (in hours) [8]		1.7 (3.3); 1.0 (2.0)		- -	- -
	Duration of caregiving (in years) [7]		17.9 (10.9); 15.5 (17.0)		- -	- -
	Number of ADL tasks (range 0–12) [11]		2.0 (2.8); 1.0 (3.0)		- -	- -
	Number of IADL tasks (range 0–10) [10]		3.6 (2.8); 3.0 (4.0)		- -	- -
	**Subjective caregiver burden**					
	Zarit Burden Interview (range 0–48) [1]		6.6 (7.0); 4.0 (9.0)		- -	- -
	**Social relationships**					
	Loneliness (UCLA-SF) (range 0–6) [4,0]		0.8 (1.3); 0.0 (1.0)		1.2 (1.4); 1.0 (2.0)	0.14 (0.03, 0.42)
	Relationship quality (QRI) (range 0–24) [1]		20.2 (3.5); 21.0 (4.0)		20.9 (3.0); 22.0 (3.0)	0.39 (0.25, 0.55)
	Social support (range 0–10) [2, 5]		6.6 (2.1); 6.0 (3.0)		7.0 (1.7); 7.0 (2.0)	0.09 (0.01, 0.48)
	Social network (SNI) [3]		3.7 (1.1); 4.0 (1.5)		4.1 (1.0); 4.0 (2.0)	0.40 (0.27, 0.56)
**6 Months**	**Social relationships**					
	Loneliness (UCLA-SF) (range 0–6) [4,0]		0.9 (1.4); 0.0 (2.0)		1.2 (1.4); 1.0 (2.0)	0.10 (0.01, 0.46)
	Relationship quality (QRI) (range 0–24) [1, 3]		20.7 (3.3); 21.0 (3.0)		20.9 (3.0); 22.0 (3.0)	0.29 (0.16, 0.47)
	Social support (range 0–10) [2, 10]		5.2 (1.7); 5.0 (1.0)		7.0 (1.7); 7.0 (2.0)	0.00 (0.00, 0.00)
	Social network (SNI) [3]		3.7 (1.1); 4.0 (1.5)		4.1 (1.0); 4.0 (2.0)	0.40 (0.27, 0.56)
**12 Months**	**Health outcomes (SF-12) (range 0–100)**					
	Mental health [4, 6]		70.8 (14.8); 70.0 (20.0)		69.3 (18.5); 70.0 (20.0)	0.24 (0.11, 0.45)
	Vitality [4]		60.5 (20.1); 60.0 (40.0)		56.0 (22.6); 60.0 (40.0)	0.13 (0.03, 0.42)
	Bodily pain intensity [4]		40.6 (31.2); 40.0 (60.0)		52.4 (29.1); 60.0 (40.0)	0.00 (0.00, 0.00)
	General health [4]		57.1 (18.8); 60.0 (20.0)		48.6 (18.9); 60.0 (20.0)	0.00 (0.00, 0.00)

*Abbreviations*: *ADL* Activities of daily living, *CI* Confidence interval, *CHF* Swiss Francs, *IADL* Instrumental activities of daily living, *ICC* Intra-class correlation, *IQR* Interquartile range, *QRI* Quality of relationship inventory, *SCI* Spinal cord injury, *SD* Standard deviation, *SNI* Social network index

#### Mediation analysis

Mediation was addressed using path analysis, which is a specific type of structural equation modelling (SEM). Social relationship constructs were included as potential mediators in the association between caregiver burden and caregiver health. Individual models were created for each caregiver health indicator and all potential mediators were entered into each model, with the addition of co-variances between all of the social relationship constructs. Bias-corrected and accelerated bootstrapping with 5000 replications with replacements was computed in order to deal with sample size and non-normality issues. Bias-corrected and accelerated bootstrapping adjusts for both bias and skewness in the bootstrap distribution. The bootstrapping technique is a technique to reduce sampling bias when variables involved in the analysis have a non-normal distribution. This also enabled the estimation of asymmetrical confidence intervals (CI) for the indirect effects in mediation analysis and for multiple mediation models, statistical support for mediation was gained if the CIs did not cross 0 [42]. In order to explore whether the *deterioration* or the *counteractive* model was empirically supported, the directionality of the paths was assessed. If the majority of paths indicated a negative relationship between subjective caregiver burden and social relationships (i.e. increased loneliness, decreased social support, relationship quality and reduced social network) then the *deterioration* model was supported, if the paths indicated a positive relationship between subjective caregiver burden and social relationships then the *counteractive* model was supported. Adequate model fit was assessed by a χ^2^ test (threshold *p* > 0.05; vulnerable to sample size), a comparative fit index (CFI) > 0.95, and the root mean square error of approximation (RMSEA) < 0.06. We report standardized regression coefficients and 95% CIs. Our sample sufficed the minimal recommended sample size [43]. Path analyses were conducted on non-imputed data using full information maximum likelihood (FIML) estimation, which adequately accounts for missing data [44]. Again, here the FIML estimation was utilised in an attempt to reduce bias resulting from item non-response. The proportion of explained variance in caregiver health was reported with the R-squared statistics.

## Results

A baseline description of the study population can be found in Table 1. The large majority of caregiving romantic partners were female (74%), with a mean age of 50.2 years, and about 70% being involved in paid work on top of their caregiving duties. In care-receivers, 60% were in paid work. Around one third of the total sample reported financial hardship. Caregiving romantic partners and care-receivers had been in formal educations for on average 14 years. In general, caregiving romantic partners provided 1.7 h a day of informal care, reported an average subjective caregiver burden score of 6.6 (range 0–48), and were on average almost 18 years in the caregiving role for their partners. Although caregiving romantic partners reported less loneliness than care-receivers (0.8 vs. 1.2), all other aspects of social relationships were reported to be of lower quality or quantity, mean social support in caregiving romantic partners was 5.2, whereas in care-receivers it was 7.0 at 6 months. Although mental health and vitality were similar within partnerships, care-receivers reported worse bodily pain and lower general health than caregiving romantic partners.

### Moderation analyses

Supplementary Table 1 shows results from unadjusted and adjusted analyses of the association between social relationships, subjective caregiver burden and health indicators, including main effects and interaction terms to test the possible moderation effect of social relationships in the caregiver burden – health association. In terms of main effects, loneliness and subjective caregiver burden showed pronounced and consistent associations with all health indicators. Relationship quality and social support decreased the negative associations of subjective caregiver burden on mental health, thus supporting the buffering model of the stress process paradigm. However, no effect moderation between caregiver burden and the health outcomes vitality, pain and general health were observed.

**Fig. 3:**
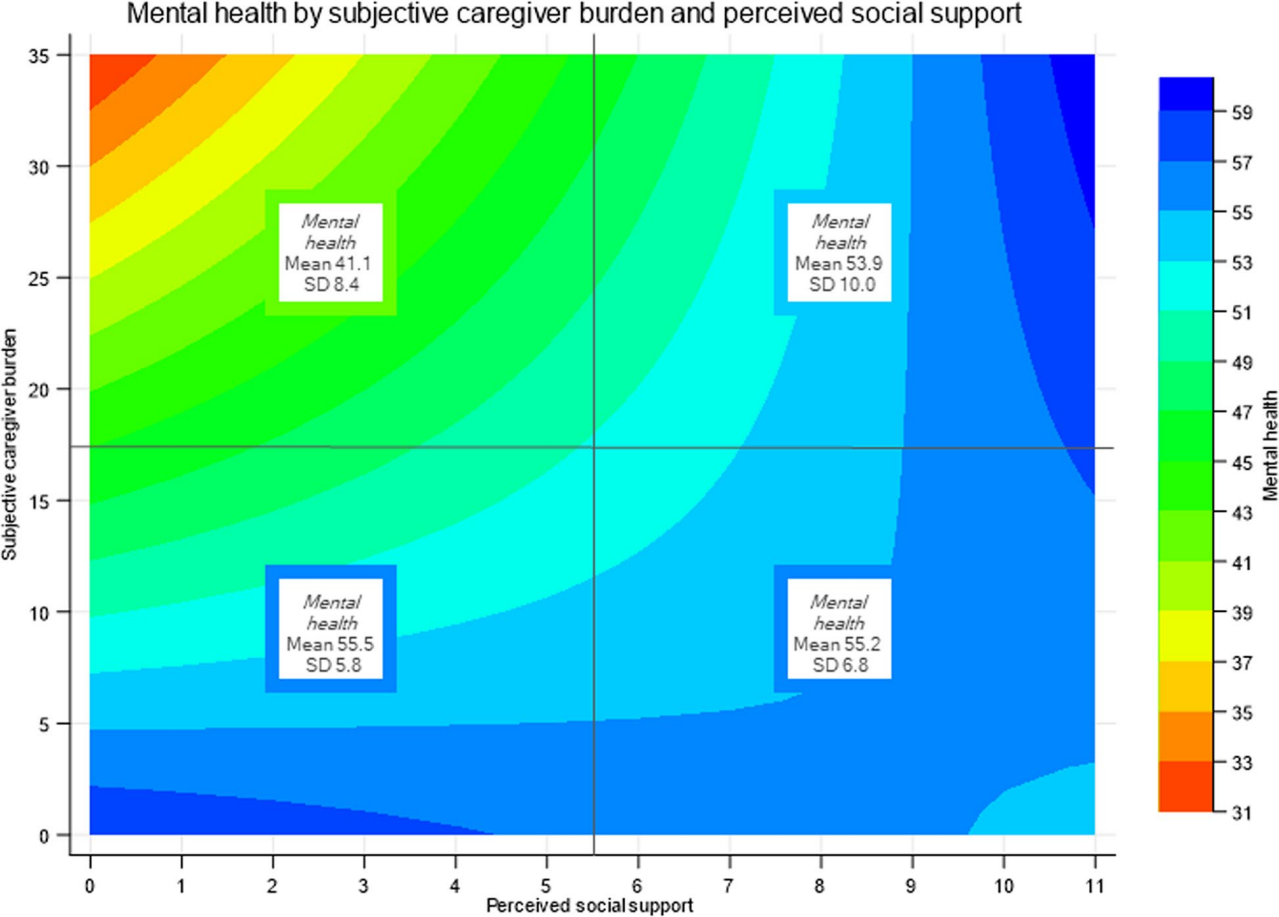
Contour plot showing the moderating effect of social support on the association between caregiver burden and mental health. Y-axis: values for predictor subjective caregiver burden. X-axis: values for moderator social support. Marginal predictions of the outcome mental health are displayed with the coloured distribution indexed on the right. To provide face validity for the marginal predictions, means and standard deviations (SD) of reported mental health are indicated in boxes. Two-by-two grouping of crude data was median-based (for social support score 5.5; for subjective caregiver burden 17.5)

**Fig. 4:**
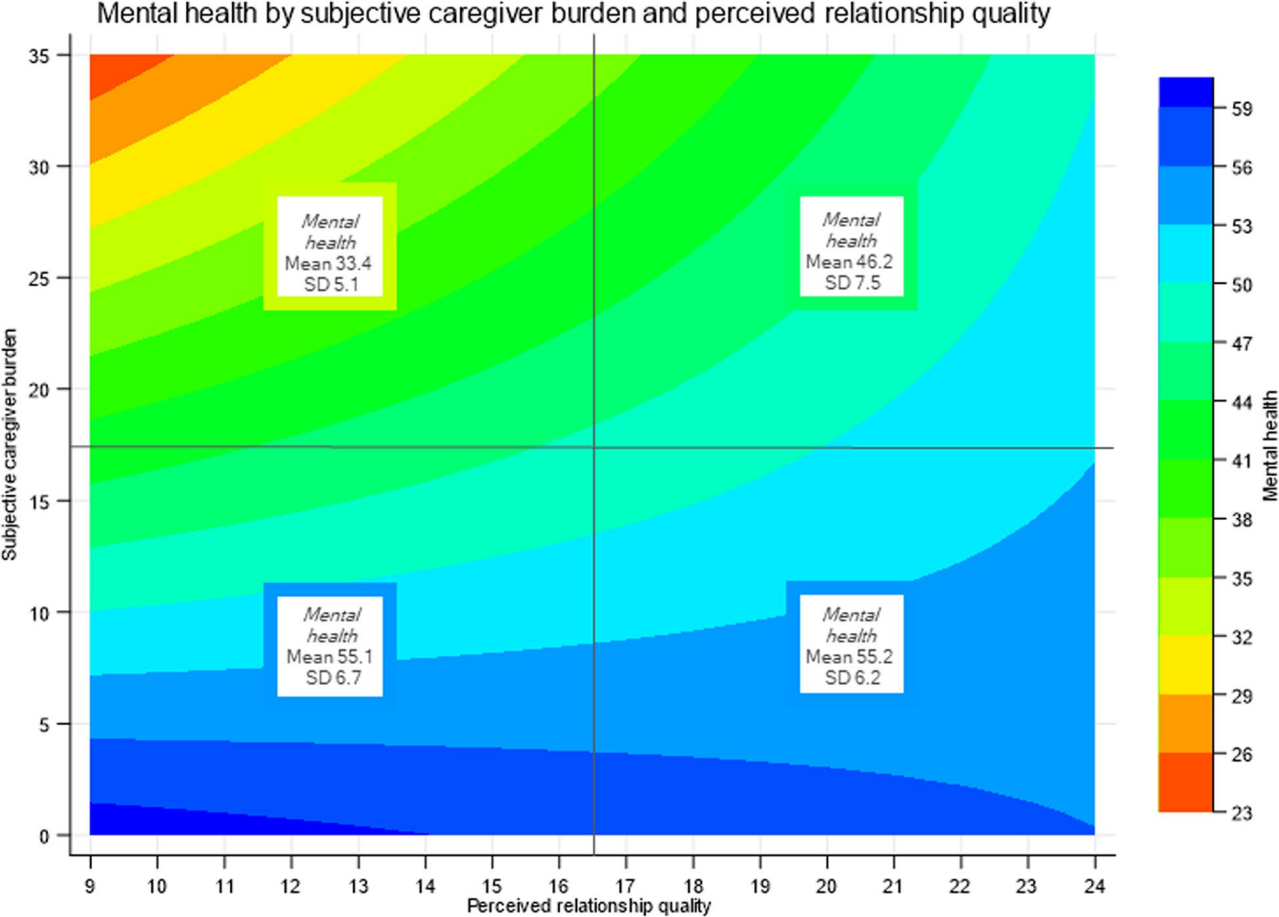
Contour plot showing the moderating effect of relationship quality on the association between caregiver burden and mental health. Y-axis: values for predictor subjective caregiver burden. X-axis: values for moderator relationship quality. Marginal predictions of the outcome mental health are displayed with the coloured distribution indexed on the right. To provide face validity for the marginal predictions, means and standard deviations (SD) of reported mental health are indicated in boxes. Two-by-two grouping of crude data was median-based (for relationship quality score 16.5; for subjective caregiver burden 17.5)

To illustrate the support for the buffering model of the stress process paradigm, marginal predictions for mental health in relation to relevant interaction terms are depicted in Figs. 3 and 4. At low levels of relationship quality or social support, mental health is largely defined by variation in subjective caregiver burden, whereas this association is progressively alleviated with an increase in relationship quality and social support. This is particularly evident in the case of social support, as mental health was essentially independent of subjective caregiver burden among caregivers reporting high social support (i.e. a value of seven or higher; Fig. 4).

### Mediation analyses

**Fig. 5:**
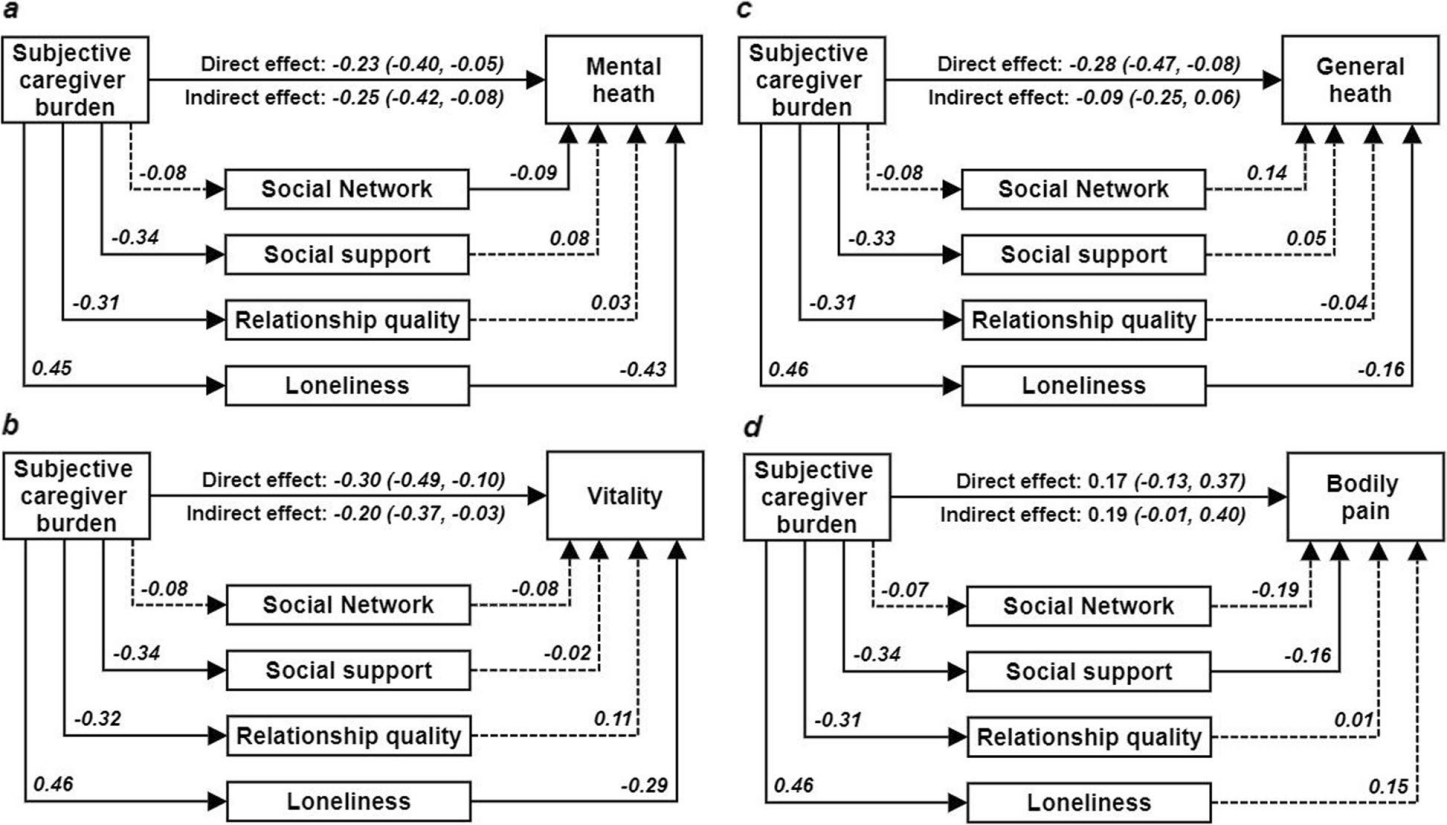
Path analyses testing mediation effects of social resources on the relationship between caregiver burden and caregiver health, showing standardised path coefficients. Bias-corrected and accelerated confidence intervals for indirect/mediation effects. Numbers on the paths indicate standardised path coefficients and dotted lines show paths where the confidence interval crosses 0 and full lines show paths where the confidence interval does not cross 0, therefore providing statistical support for the relevance of the path

Figure 5 presents the path analyses testing the mediation effects of social relationships on the pathway between caregiver burden and health by assessing the indirect effect and the corresponding bias corrected and accelerated CIs. Mediating effects of social relationships were supported for mental health (indirect effect -0.25, CI -0.42- -0.08) and vitality (indirect effect -0.20, CI -0.37- -0.03; (Fig. 5a and b), where the proportion of mediated effect was 52% and 40% respectively. Models suggest that the mediating effects were exerted through caregiver burden's positive association with loneliness, and subsequently loneliness's detrimental effect on mental health and vitality. In the majority of models, the direction of the paths indicated a negative association between caregiver burden and social relationships, lending therefore support for the *deterioration* model of the stress process paradigm.

## Discussion

Our study is the first to untangle the relationships between caregiver burden, different social relationship concepts and caregiver health by testing different models of the stress process paradigm. Our findings provide support for the *buffering* model, as social support and partner relationship quality were found to modify the negative association of caregiver burden with mental health, however this did not hold true for other health indicators. The *deterioration* model was supported for mental health and vitality due to the negative association between caregiver burden and social relationships. In particular loneliness played an important role as high levels of burden were associated with high levels of loneliness, and subsequently there was a strong association between loneliness and poor mental health and vitality. While we observed consistent findings supporting the *buffering* and *deterioration* models concerning mental health, this did not hold true for indicators of physical or general health.

Although there is a vast amount of research exploring the determinants of caregiver burden [45], and how the stressors of caregiving impact upon health [1, 3, 46], there is relatively little research exploring how different psychosocial resources can enable caregivers to protect their health [14, 47]. Our study findings corroborate the results of classical stress buffering studies [11, 13, 48], as we find evidence to support the assumption that the detrimental physiological and emotional stress reactions to high subjective caregiver burden [2, 3] are reduced, i.e. buffered, if the caregiver perceives their relationship to the care-receiver as high quality and supportive. Although our study cannot provide causal evidence for the buffering effects of social relationships, our results suggest a level of effect modification by relationship quality and social support. Our study therefore highlights the potential of social support and relationship quality to override the negative consequences of caregiver burden on mental health and therefore provides evidence for the targeting of the qualitative aspects of social relationships by interventions addressing caregiver health. These results suggest that if caregivers are able to enhance the quality and supportive nature of their relationships to a certain desired level, in the case of our results a value of over seven for social support, the negative effects of caregiver burden diminish considerably. In line with current literature [10, 49], this study therefore highlights the importance of the qualitative aspects of social relationships in protecting caregiver's mental health. In comparison quantitative aspects, such as social network size was weakly related to caregiver health indicators, and had no interaction effects with subjective burden. This finding might be explained by the fact that quantitative characteristics of social relationships (i.e. membership in clubs, number of close persons) are not decisive for coping with adversity. It is rather the resources provided by these relationships, which may enable an individual to cope with stress by facilitating reappraisal of a stressful situation, providing practical solutions, or directly influencing the psychological or physiological stress processes, which are detrimental to health [48].

In addition to the findings on the buffering effects of functional aspects of social relationships, we also found evidence to support the deterioration model of the stress process paradigm. Our study found that increased caregiver burden was linked to the deterioration of social relationships, as those with higher levels of burden at baseline reported lower levels of relationship quality and social support, and higher levels of loneliness at 6 months. Caregiver burden is often thought to be a consequence of poor access to social resources, however it is also plausible that the caregiver role is damaging to social relationships [17, 18]. The care-receiver/caregiver relationship can suffer as a result of caregiving duties, the emotional and psychological strain of receiving and providing care, and the unequal balance of emotional and instrumental support provided in the relationship [50]. This may also be extended to a wider social network if caregiving activities restrict the participation in, and maintenance of, other social relationships. Therefore, the detrimental effect of burden on social relationships had a further knock-on effect on health, this is in addition to the already harmful direct effect which caregiver burden has on health [2].

It is somewhat surprising that no mediation or moderation effects of social relationships in relation to general health and bodily pain were detected, especially as subjective caregiver burden had a relevant main effect on general health and bodily pain, comparable to the main effects of mental health and vitality. We do however see some evidence that loneliness may lie on the pathway between caregiver burden and general health, and loneliness does indeed have main effects on all health outcomes and has been seen to play a prominent role in determining health [51]. It is also conceivable that health conditions that are often associated with physiological stress responses, such as cardiovascular disease, have a long disease progression. This may not have been captured, as we have no information on the chronicity of stressful caregiver burden.

### Strengths and limitations

This study is the first to test three theoretical models of the stress process paradigm and the strong theoretical basis of the models tested presents a major strength of this study. Furthermore, we were able to investigate different quantitative and qualitative aspects of social relationships and their associations with multiple health indicators. All associations were tested using multivariate models, which considered relevant confounders, and contemporary techniques were applied to test mediation and moderation models using longitudinal data. Furthermore, the pro-WELL study was nested in a large cohort study, showing good representation of the source population of care-receivers in terms of basic characteristics [30] indicating that gender, lesion characteristics (severity, etiology), and general well-being was not systematically different in source study and nested study participants [30]. However, pro-WELL participants were more likely to be involved in paid work than in the source population and we cannot exclude that this overrepresentation has an impact on constructs included in this study and on the generalisability of results.

Yet, a possible limitation includes volunteer bias with respect to caregiver burden or associated health status, as the couples least burdened by the caregiver situation may have been more likely to participate than couples with high caregiver burden. Moreover, the self-report nature of variables may have been subject to recall bias and social desirability, especially variables concerning partnership quality as both members of the couples participated. In addition, although we used longitudinal data we were prohibited from carrying out a more sophisticated analysis due to the fact the caregiver burden was only measured at baseline and we could therefore not factor in the dynamic nature of burden over time. In addition, longitudinal inference following regression analysis may have been subject to bias due to unmeasured confounding, particularly due to unmeasured personal and psychological characteristics of respondents. Moreover, the limited sample of the study may challenge the internal validity of the study, as limiting the power to identify underlying associations as well as measurement precision. Furthermore, due to our relatively limited sample size, sophisticated modelling techniques which assess the competing effects of moderation and mediation in one model were not possible. This therefore prevented the testing of mediated moderation, or moderated mediation. This also means that our results on moderating effect do not rule out the possibility of mediation, and vice versa. Further, we cannot evaluate whether the use of different response modes had an impact on response behaviour. In addition, in order to reduce complexity in our models' potential confounders were only introduced at baseline. It is presumed that many of the associations tested are reciprocal in nature and therefore a causal effect cannot be specifically stated. We would therefore encourage the use of life course data on the caregiver experience in order to unravel complex phenomenon over time. Finally, inherent to the use of self-reported data, regression-based inference of associations may have been induced by latent psychological personal factors that were not accounted for, despite the multivariable adjustment employed in the present study.

### Practical implications

This study highlights the potential of social support and relationship quality to override the negative consequences of caregiver burden on mental health, indicating that interventions targeting qualitative aspects of social relationships present a promising strategy to support mental health of highly burdened caregivers. Several studies demonstrated that caregivers express a wish for more interaction with others, either to directly address issues of social isolation, or to benefit from training or education from service providers. This could be offered in the form of peer support, whereby both factual information and emotional support could be shared [45, 52, 53]. This could present a good opportunity to not only address issues concerning burden and strain, but also the often associated issues of partner relationship quality, mutuality and social support. Social support interventions are often criticized for encouraging or manufacturing, "synthetic" support [11], but in this case interventions may provide an opportunity to strengthening existing relationships, whilst also working on relationship issues which are a direct result of the caregiving dynamic [54]. Cognitive behavioural therapy has been shown to improve perceived social support [55] and is described as a promising approach to intervene on maladaptive social cognitions in relation to feelings of loneliness or low belongingness which may be a direct result of caregiving [56]. Therefore, interventions, which are aimed at improving the dyadic relationship between caregiver and care-receiver, whilst also addressing practical elements of the caregiving dynamic, and providing additional emotional and tangible support, would have the potential to improve relationships, reduce burden and have the additive effect of improving caregiver's health.

## Conclusions

Our findings provide support for the *buffering* and *deterioration* models of the stress process paradigm, whereby social support and relationship quality were found to modify the negative associations of caregiver burden with mental health, and social relationships were found to deteriorate with ever greater caregiver burden. Our study highlights the potential of social support and relationship quality to override the negative consequences of caregiver burden on mental health, and therefore provides evidence to support the targeting of qualitative aspects of social relationships by interventions addressing caregiver health. Interventions, which are aimed at improving the dyadic relationship between caregiver and care-receiver, whilst also addressing practical elements of the caregiving dynamic, would have the potential to improve relationships, increase perceived social support and have the additive effect of improving caregiver's health, especially in those individuals where caregiver burden is high.

## Supplementary Information

**Additional file 1:** **Supplementary Table 1.** Moderation effects of social resources in the relationship between caregiver burden and caregiver health.

## Data Availability

The datasets used and analysed during the current study is available on DRYAD at https://doi.org/10.5061/dryad.02v6wwq4b.

## Abbreviations

ADL: Activities of daily living
CI: Confidence interval
CHF: Swiss Francs
DAG: Directed acyclic graph
FIML: Full information maximum likelihood
IADL: Instrumental activities of daily living
ICC: Intra-class correlation
IQR: Interquartile range
MICE: Multiple imputation using chained equations;
QRI: Quality of relationship inventory
SCI: Spinal cord injury
SEM: Structural Equation Modelling
SD: Standard deviation
SNI: Social network index
ZBI: Zarit Burden Interview
