# Supplementary material for: Untangling the role of social relationships in the association between caregiver burden and caregiver health: an observational study exploring three coping models of the stress process paradigm
Source: BMC Public Health. 2022 Sep 13;22:1737. doi: 10.1186/s12889-022-14127-3 (PMC9472370; doi:10.1186/s12889-022-14127-3)
Supplement: Supplementary file 1 — Additional file 1: Supplementary Table 1. Moderation effects of social resources in the relationship between caregiver burden and caregiver health. [file 12889_2022_14127_MOESM1_ESM.docx]

**Supplementary Table 1: Moderation effects of social resources in the relationship between caregiver burden and caregiver health**

|  | **Mental health** | | **Vitality** | | **Bodily pain** | | **General health** | |
| --- | --- | --- | --- | --- | --- | --- | --- | --- |
|  | **Coefficient**  **(95% CI)** | ***p value*** | **Coefficient**  **(95% CI)** | ***p value*** | **Coefficient**  **(95% CI)** | ***p value*** | **Coefficient**  **(95% CI)** | ***p value*** |
| **Main Effects** | | | | | | | | |
| **Loneliness** |  |  |  |  |  |  |  |  |
| Model 1 | -2.77 (-3.68--1.85) | *<0.001* | -2.64 (-3.77--1.52) | *<0.001* | 0.76 (-0.77-2.29) | *0.328* | -1.16 (-2.24--0.08) | *0.035* |
| Model 2 | -1.97 (-2.89--1.06) | *<0.001* | -2.10 (-3.33--0.88) | *0.001* | 0.77 (-0.79-2.33) | *0.332* | -0.56 (-1.68-0.55) | *0.322* |
| Model 3 | -1.87 (-2.82--0.92) | *<0.001* | -2.03 (-3.30--0.77) | *0.002* | 1.16 (-0.53-2.85) | *0.178* | -1.07 (-2.26-0.12) | *0.078* |
| **Relationship quality** |  |  |  |  |  |  |  |  |
| Model 1 | 0.73 (0.35-1.11) | *<0.001* | 0.93 (0.50-1.36) | *<0.001* | -0.28 (-0.88-0.32) | *0.360* | 0.22 (-0.21-0.64) | *0.319* |
| Model 2 | 0.17 (-0.19-0.54) | *0.348* | 0.38 (-0.08-0.84) | *0.101* | -0.01 (-0.61-0.58) | *0.962* | -0.11 (-0.53-0.31) | *0.603* |
| Model 3 | 0.08 (-0.28-0.45) | *0.649* | 0.34 (-0.13-0.81) | *0.151* | -0.04 (-0.66-0.58) | *0.896* | -0.12 (-0.55-0.31) | *0.583* |
| **Social support** |  |  |  |  |  |  |  |  |
| Model 1 | -0.39 (-1.02-0.23) | *0.216* | -0.31 (-1.09-0.46) | *0.424* | -0.21 (-1.14-0.73) | *0.664* | 0.17 (-0.53-0.88) | *0.626* |
| Model 2 | 0.28 (-0.47-1.04) | *0.460* | 0.06 (-0.91-1.03) | *0.900* | -0.36 (-1.61-0.89) | *0.569* | -0.01 (-0.88-0.86) | *0.983* |
| Model 3 | 0.22 (-0.59-1.03) | *0.586* | -0.26 (-1.29-0.77) | *0.615* | -0.40 (-1.75-0.96) | *0.565* | 0.22 (-0.71-1.14) | *0.641* |
| **Social network** |  |  |  |  |  |  |  |  |
| Model 1 | 0.19 (-0.98-1.36) | *0.748* | 0.26 (-1.17-1.68) | *0.722* | -1.13 (-2.85-0.59) | *0.196* | 1.22 (-0.02-2.45) | *0.054* |
| Model 2 | 0.03 (-1.03-1.08) | *0.961* | 0.09 (-1.20-1.38) | *0.892* | -0.97 (-2.63-0.68) | *0.247* | 0.91 (-0.27-2.09) | *0.129* |
| Model 3 | -0.17 (-1.23-0.89) | *0.751* | -0.05 (-1.39-1.29) | *0.940* | -1.18 (-2.91-0.56) | *0.182* | 1.05 (-0.18-2.27) | *0.095* |
|  |  |  |  |  |  |  |  |  |
| **Subjective caregiver burden (ZBI)** | |  |  |  |  |  |  |  |
| Model 1 | -0.48 (-0.65--0.30) | *<0.001* | -0.54 (-0.75--0.33) | *<0.001* | 0.48 (0.20-0.76) | *0.001* | -0.43 (-0.63--0.24) | *<0.001* |
|  |  |  |  |  |  |  |  |  |
| Model 2^a^ | -0.21 (-0.41--0.01) | *0.037* | -0.42 (-0.67--0.16) | *0.002* | 0.35 (0.02-0.69) | *0.041* | -0.36 (-0.60--0.12) | *0.004* |
| Model 3^a^ | -0.23 (-0.43--0.03) | *0.022* | -0.44 (-0.70--0.18) | *0.001* | 0.26 (-0.08-0.61) | *0.136* | -0.28 (-0.52--0.04) | *0.025* |
|  |  |  |  |  |  |  |  |  |
| Model 2^b^ | -0.34 (-0.53--0.15) | *<0.001* | -0.42 (-0.65--0.19) | *0.001* | 0.43 (0.13-0.73) | *0.005* | -0.39 (-0.61--0.18) | *0.001* |
| Model 3^b^ | -0.37 (-0.55--0.19) | *<0.001* | -0.46 (-0.69--0.23) | *<0.001* | 0.38 (0.07-0.69) | *0.017* | -0.36 (-0.58--0.14) | *0.002* |
|  |  |  |  |  |  |  |  |  |
| Model 2^c^ | -0.27 (-0.48--0.06) | *0.013* | -0.42 (-0.68--0.15) | *0.002* | 0.38 (0.04-0.71) | *0.030* | -0.40 (-0.65--0.16) | *0.002* |
| Model 3^c^ | -0.29 (-0.50--0.08) | *0.007* | -0.47 (-0.74--0.20) | *0.001* | 0.28 (-0.08-0.63) | *0.127* | -0.33 (-0.58--0.08) | *0.012* |
|  |  |  |  |  |  |  |  |  |
| Model 2^d^ | -0.44 (-0.61--0.26) | *<0.001* | -0.51 (-0.74--0.29) | *<0.001* | 0.47 (0.19-0.75) | *0.001* | -0.48 (-0.68--0.28) | *<0.001* |
| Model 3^d^ | -0.44 (-0.62--0.26) | *<0.001* | -0.54 (-0.77--0.31) | *<0.001* | 0.39 (0.09-0.69) | *0.010* | -0.43 (-0.64--0.22) | *<0.001* |
|  |  |  |  |  |  |  |  |  |
| **Interaction effects** | | | | | | | | |
|  | | | | | | | | |
| **Loneliness x ZBI** |  |  |  |  |  |  |  |  |
| Model 2 | -0.05 (-0.13-0.02) | *0.179* | 0.04 (-0.06-0.14) | *0.399* | 0.04 (-0.09-0.17) | *0.576* | -0.02 (-0.12-0.07) | *0.651* |
| Model 3 | -0.05 (-0.13-0.02) | *0.175* | 0.05 (-0.05-0.15) | *0.347* | 0.03 (-0.11-0.16) | *0.693* | -0.03 (-0.12-0.07) | *0.590* |
| **Relationship quality x ZBI** | |  |  |  |  |  |  |  |
| Model 2 | 0.06 (0.02-0.10) | *0.005* | 0.04 (-0.01-0.09) | *0.154* | -0.03 (-0.10-0.04) | *0.400* | 0.04 (-0.01-0.09) | *0.148* |
| Model 3 | 0.06 (0.01-0.10) | *0.009* | 0.02 (-0.03-0.08) | *0.382* | -0.01 (-0.09-0.06) | *0.702* | 0.05 (-0.01-0.10) | *0.086* |
| **Social support x ZBI** |  |  |  |  |  |  |  |  |
| Model 2 | 0.10 (0.03-0.17) | *0.007* | 0.06 (-0.03-0.15) | *0.164* | -0.04 (-0.15-0.07) | *0.482* | 0.02 (-0.06-0.10) | *0.601* |
| Model 3 | 0.09 (0.02-0.16) | *0.013* | 0.05 (-0.04-0.14) | *0.245* | -0.05 (-0.17-0.06) | *0.370* | 0.04 (-0.05-0.12) | *0.396* |
| **Social network x ZBI** |  |  |  |  |  |  |  |  |
| Model 2 | 0.12 (-0.04-0.29) | *0.144* | 0.08 (-0.12-0.29) | *0.431* | -0.01 (-0.26-0.24) | *0.943* | -0.14 (-0.32-0.05) | *0.149* |
| Model 3 | 0.10 (-0.07-0.26) | *0.242* | 0.04 (-0.17-0.26) | *0.706* | -0.02 (-0.29-0.25) | *0.881* | -0.07 (-0.26-0.13) | *0.505* |

All social relationship constructs were entered individually into the models due to the restricted sample size.

Model 1 = regression model including only main effects; Model 2 = regression model containing main effects and interaction effects; Model 3 = regression model including main effects, interaction effects and the confounders: age, language, sex, lesion characteristics of care receiver, duration of caregiving, financial hardship, employment status.

1. Model including social relationship construct loneliness
2. Model including social relationship construct relationship quality
3. Model including social relationship construct social support
4. Model including social relationship construct social network

Abbreviations: CI: Confidence interval.
